# Association of Constipation with risk of end-stage renal disease in patients with chronic kidney disease

**DOI:** 10.1186/s12882-019-1481-0

**Published:** 2019-08-05

**Authors:** Chung-Yen Lu, Yin-Cheng Chen, Yu-Wen Lu, Chih-Hsin Muo, Ray-E Chang

**Affiliations:** 1 Department of Sport and Health Management, Da-Yeh University, Changhua, 515 Taiwan; 2 Department of Chinese Medicine, China Medical University Hospital, Taipei Branch, Taipei, 114 Taiwan; 3 Department of Chinese Medicine, Show Chwan Memorial Hospital, Changhua, 500 Taiwan; 4 Department of Internal Medicine, Taipei Hospital, Ministry of Health and Welfare, New Taipei, 242 Taiwan; 5 Institute of Health Policy and Management, National Taiwan University, No 17, Xu-Zhou Rd, Rm 639, Taipei, 100 Taiwan; 6 Department of Chinese Medicine, Chang Bing Show Chwan Memorial Hospital, Changhua, 505 Taiwan; 7 Management Office for Health Data, China Medical University Hospital, Taichung, 404 Taiwan

**Keywords:** Kidney-gut axis, Chronic kidney disease, Gut microbiota, Constipation, End-stage renal disease

## Abstract

**Background:**

Chronic Kidney Disease (CKD) is a growing public health problem. Many risk factors were identified and interventions were applied accordingly, but the incidence of end-stage renal disease continued increasing. Some other risk factors may be ignored. Gut microbiota has been recognized as an important endogenous organ. The kidney-gut axis would contribute to gut dysbiosis, which might worsen CKD. Constipation, commonly seen in CKD, was one of the clinical presentation of gut dysbiosis. The clinical impact of constipation to CKD remains unknown. Our study aimed at assessing the risk of ESRD between CKD patients with and without constipation in a nationwide database.

**Methods:**

We identified newly diagnosed cases of CKD without constipation history before in 2000–2011 from the Taiwan National Health Insurance database. Subjects who developed constipation later formed constipation group. The others without constipation matched by propensity score formed non-constipation group. The incidence rates and hazards of ESRD in patients with and without constipation by the end of 2013 were compared by using Cox proportional hazard models with a time-dependent variable.

**Results:**

The incidences of ESRD per 1000 person-years were 22.9 for constipation group and 12.2 for non-constipation group, respectively. Cox proportional hazard models with a time-dependent variable revealed an adjusted hazard ratio of 1.90 (95% CI, 1.60–2.27). Compared to the CKD patients without constipation, adjusted hazard ratio for the CKD patients with laxatives < 33, 33–197 and ≥ 198 days per year were 0.45 (0.31–0.63), 1.85 (1.47–2.31) and 4.41 (3.61–5.39) respectively.

**Conclusion:**

In a population of newly-diagnosed CKD patients, we observed that subjects with de novo constipation, as compared with non-constipation, have increased risk of developing ESRD. More severe constipation would increase the risk further.

**Electronic supplementary material:**

The online version of this article (10.1186/s12882-019-1481-0) contains supplementary material, which is available to authorized users.

## Background

Chronic kidney disease (CKD) is a global health issue. If CKD couldn’t be adequately controlled, it would progress to end-stage renal disease (ESRD), which requires costly intervention of renal replacement therapy and will be a great burden to family and government [1]. There are many risk factors for CKD, including diabetes mellitus, hypertension, analgesics, herbs, kidney stones, and infection, etc. [2–8]. However, even under strict control, CKD may still progress. Some other unknown risk factors may be ignored.

In the near decades, human gut was found to harbor 10^11^ to 10^12^ microbiota which influence the nutrition, metabolism, and immune function of the host and has been recognized as an endogenous organ inside the body [9–12]. In healthy people, the gut microbiota interact harmoniously with the host in mutualistic relationship, so called symbiosis. However, many chronic diseases will disturb the gut microbiome and turn symbiosis to dysbiosis, which was related to some complications and the worsening of the chronic disease [13]. The interaction between kidney and gut, so called kidney-gut axis, was one of the concerned [14]. In the “kidney-to-gut” side, CKD is associated with fluid overload with intestinal wall edema, accumulation of uremic toxin, decreased consumption of dietary fibers, oral use of phosphate binders and/or iron, and so on. These factors all contribute to intestinal dysbiosis [12, 15]. Constipation is the most common gut abnormality in CKD patients. In the opposite “gut-to-kidney” side, gut microbiota also produce some uremia retention molecules, like indoxyl sulfate and p-cresyl sulfate, which are fully excreted by the normal kidney but would accumulate as kidney fails [16]. The increased levels of these metabolites are associated with chronic inflammation, increased cardiovascular mortality and the progression of CKD [17–19]. The interaction between kidney and gut would be a vicious cycle that worsen the kidney further. Constipation might be an indicator of gut dysbiosis in CKD patients and suggested an ongoing vicious cycle. However, the constipation in CKD patients was usually ignored and not even thought as a risk factor of the progression of CKD.

Whether the CKD patients with constipation had poor profiles of gut microbiome inside was not known yet. We hypothesized CKD patients with constipation may suffer from rapid progression of kidney disease for the negative effects of poor profiles of gut microbiome. The current study tried to explore the relationship between CKD progression and constipation by analyzing a nationwide database.

## Methods

### Data source

We used the Longitudinal Health Insurance Database (LHID) 2000 [20], a medical claims database that includes one million subjects randomly selected from among people insured during 1996–2000 by the National Health Insurance (NHI) of Taiwan, to design the population-based retrospective cohort study. 96% of the total 23 million Taiwan’s residents were covered by the healthcare of NHI by the end of 1996 [21] and more than 99% by 2011 [22]. The LHID was for research purposes and represented the general population by age and sex distributions and included multiple claims files, like the expenditures and orders of ambulatory and inpatient care, the prescriptions of drugs, and registry for beneficiaries, which altogether provide information about the diagnoses and details of the utilization of healthcare. The diagnostic codes of the claims are recorded according to the International Classification of Diseases, 9th Revision, Clinical Modification (ICD-9-CM). According to Personal Information Protection Act, the identification of insurant was re-coded and this study was approved by the Institutional Review Board of the China Medical University Hospital (CMUH104-REC2–115(CR-1)) in Taiwan.

### Study population

**Fig. 1:**
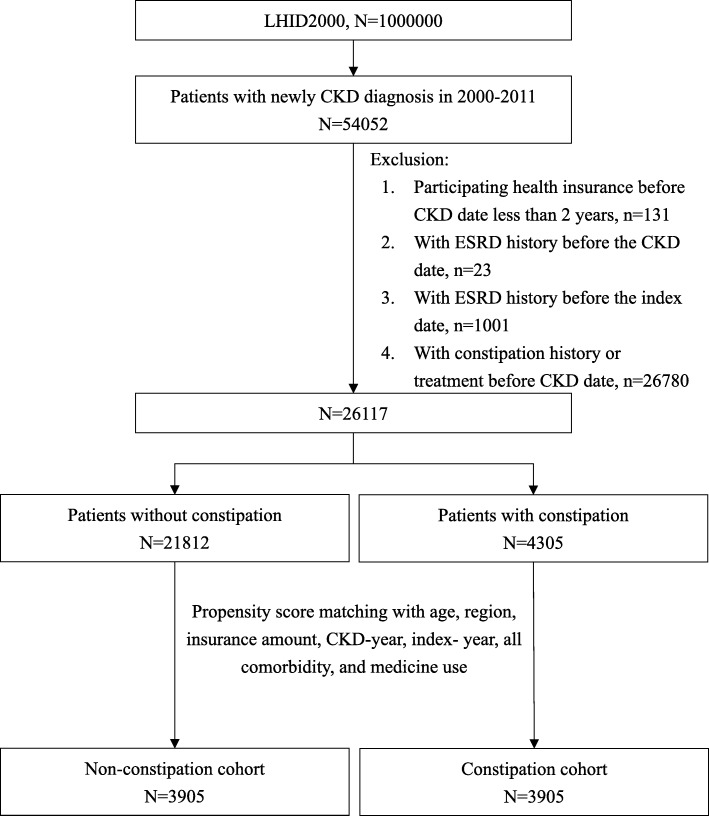
Flow chat

Within LHID 2000, those more than 20 years old and with new CKD diagnosis (CKD related ICD-9-CM codes shown in Additional file 1: Table S1) in at least three outpatient claims between 1 January 2000 and 31 December 2011 were collected as study population (*n* = 54052). The date of the third CKD diagnosis was the CKD date. The date of the diagnosis of constipation was the index date (detailed definition seen “Exposures to constipation”). 131 subjects participating health insurance less than 2 years before the CKD date were excluded. 23 subjects with ESRD diagnosis any time before the CKD date, and 1001 subjects with ESRD diagnosis any time before the index date, were excluded. Finally, 26780 patients with constipation history before their index date were excluded. A total of 26117 subjects were included in the data analysis (Fig. 1).

### Exposures to constipation

We defined constipation group as patients who had at least two claims for constipation (ICD-9-CM 564.0x) or Laxatives prescription (ACT code A06AA, A06AB, A06AC, A06AD, A06AG, A06AH or A06AX) over 60 days apart within one year before their index date. The date of their second visit of a constipation outpatient clinic was the index date. In the cohort, those with constipation were 4035 and without constipation was 18797. The constipation group and non-constipation group, each consisting of 3909 subjects, were formed after matching by propensity score according to age, region, insurance salary, CKD-year, index year, concomitant diseases, and related medications (Fig. 1). Among constipation group, we further divided them into subgroups according to the duration of laxative use. For there were no insights or references about the relationships between the duration of constipation and ESRD, we thus divided the constipation group into 3 equal subgroups by the numbers of the subjects, which may represent mild, moderate, and severe constipation respectively, were done. The cut-off time was the lower third: < 33, the middle third: 33–197 and the upper third: ≥198 days per year. Besides, the different numbers of types (less than or equal to one type (≤ 1 type) and more than one type (> 1 type)) of laxatives used were compared too.

### Patient characteristics

The demographic variables included age, sex, geographic region and urbanization level [23]. Two kinds of concomitant diseases were adopted. The first was the comorbidities related to the progression of CKD, including diabetes mellitus, hypertension, hyperlipidemia, acute coronary syndrome, cerebrovascular disease and chronic obstructive pulmonary disease. The second was the diseases predisposing constipation, including gastrointestinal tract malignancy, inflammatory bowel disease, hypothyroidism, Parkinson’s disease, and mytonic dystrophy. (Concomitant diseases ICD-9-CM codes shown in supplementary Table S1). Besides, these concomitant diseases were considered to be present if the diagnosis codes were recorded on at least one inpatient claim or two outpatient claims in the two years before the index date. Also two kinds of medications were adopted. The first was the medication which may cause nephrotoxicity, including non-steroid anti-inflammatory drugs (NSAIDs) and analgesics other than NSAIDs. The second was the medication which may cause constipation, including opioids, aluminum antacids, antidepressants, antihistamines, antispasmodics, anticonvulsants, antiarrythmics, anti-diarrheals, 5-HT3 receptor antagonists, beta-adrenoceptor antagonists, calcium channel blockers, diuretics, calcium and iron supplement (Related medications ATC classification system codes shown in Additional file 1: Table S2).

### Outcome variable

The study end point was ESRD, which was defined by CKD plus chronic renal failure under regular dialysis (ICD-9-CM codes 585) from the registry for catastrophic illness patient database in Taiwan NHI. ESRD rate = ESRD events numbers / 1000 person-years. The ESRD rate in non-constipation group was the reference. The ESRD rate in the other groups was compared to the non-constipation.

### Statistical analysis

The baseline characteristics of constipation group and non-constipation group were summarized using descriptive statistics. The follow-up duration started at the index date and ended at the occurrence of ESRD, withdrawal from NHI program, or the termination of this study on 31 December 2013, whichever came first. To account for the immortal time of constipation patients, we assessed the association between constipation and risk of developing ESRD using Cox proportional hazard models with a time-dependent variable for constipation group. For people in constipation group, their follow-up person-time were exactly classified into the non-constipation group, before the index date of the constipation, and the constipation group, after the index date. The Cox models yielded the hazard ratio (HR) and 95% confidence interval (CI) for ESRD in constipation group, with non-constipation group as the reference group. All statistical analyses were performed using SAS 9.4 software for windows (SAS Institute, Cary, NC, USA) and the significant level was set at 0.05 in two-tailed test.

## Results

The demographics, socioeconomic status, concomitant diseases and related medications between the two groups with and without constipation were presented in Table 1. Among the 7818 newly diagnosed cases of CKD, 3909, or 50%, had constipation and the other 50% had no constipation during the follow-up time. Table 1 presented the patients’ characteristics in two years before index date. Data for demographics, socioeconomic status, concomitant diseases and related medications showed no difference in distribution between groups.

**Table 1:** Patients’ characteristics

Variable	Overall		Constipation		Non-constipation		*p*-value
Patient no.	*N* = 7818		*N* = 3909		N = 3909		
Age, years (S.D.)	65.2	(14.3)	65.0	(15.0)	65.4	(13.5)	0.20
Female (%)	3540	(45.3)	1766	(45.2)	1774	(45.4)	0.86
Insurance amount, NTD (%)							0.77
Fixed premium or dependent	1519	(19.4)	765	(19.6)	754	(19.3)	
< 20,000	3276	(41.9)	1623	(41.5)	1653	(42.3)	
20,000–39,999	2391	(30.6)	1194	(30.5)	1197	(30.6)	
≧39,999	632	(8.08)	327	(8.37)	305	(7.80)	
Region (%)							0.87
North	2779	((35.6)	1386	(35.5)	1393	(35.6)	
Center	1710	(21.9)	856	(21.9)	854	(21.9)	
South	2608	(33.4)	1296	(33.2)	1312	(33.6)	
East	721	(9.22)	371	(9.49)	350	(8.95)	
Urbanization (%)							0.88
Urban	4403	(56.3)	2212	(56.6)	2191	(56.1)	
Satellite	2529	(32.4)	1259	(32.2)	1270	(32.5)	
Rural	886	(11.3)	438	(11.2)	448	(11.5)	
Comorbidities (%)							
Acute coronary syndrome	371	(4.75)	188	(4.81)	183	(4.68)	0.79
Diabetes	3735	(47.8)	1862	(47.6)	1873	(47.9)	0.80
Hypertension	5277	(67.5)	2623	(67.1)	2654	(67.9)	0.45
Hyperlipidemia	2919	(37.3)	1452	(37.2)	1467	(37.5)	0.73
COPD	1046	(13.4)	516	(13.2)	530	(13.6)	0.64
Cerebrovascular disease	631	(8.07)	343	(8.77)	288	(7.37)	0.02
Gastrointestinal tract cancer	227	(2.90)	113	(2.89)	114	(2.92)	0.95
Inflammatory bowel disease	68	(0.87)	31	(0.79)	37	(0.95)	0.46
Hypothyroidism	101	(1.29)	53	(1.36)	48	(1.23)	0.62
Parkinson’s disease	184	(2.35)	100	(2.56)	84	(2.15)	0.23
Mytonic dystophy	0	(0.00)	0	(0.00)	0	(0.00)	
Medications (%)							
NSAIDs	6271	(80.2)	3121	(79.8)	3150	(80.6)	0.41
Analgesic drugs other than NSAIDs	6429	(82.2)	3210	(82.1)	3219	(82.4)	0.79
Aluminum antacids	873	(11.2)	424	(10.9)	449	(11.5)	0.37
Antiarrythmics	471	(6.02)	237	(6.06)	234	(5.99)	0.89
Anticonvulsants	1052	(13.5)	537	(13.7)	515	(13.2)	0.47
Antidepressants	1271	(16.3)	646	(16.5)	625	(16.0)	0.52
Antidiarrheal	1155	(14.8)	578	(14.8)	577	(14.8)	0.97
Antihistamines	5590	(71.5)	2775	(71.0)	2815	(72.0)	0.32
Antispasmodics	2780	(35.6)	1373	(35.1)	1407	(36.0)	0.42
Beta-blockers	3277	(41.9)	1627	(41.6)	1650	(42.2)	0.60
Calcium channel blockers	4390	(56.2)	2197	(56.2)	2193	(56.1)	0.93
Calcium supplement	986	(12.6)	493	(12.6)	493	(12.6)	> 0.99
Diuretics	3408	(43.6)	1694	(43.3)	1714	(43.9)	0.65
Iron supplement	568	(7.27)	279	(7.14)	289	(7.39)	0.66
Opioids	1630	(20.9)	821	(21.0)	809	(20.7)	0.74
Serotonin (5HT3) antagonists	95	(1.22)	43	(1.10)	52	(1.33)	0.35
Propensity score, (S.D.)	0.30	(0.19)	0.30	(0.19)	0.30	(0.19)	> 0.99

Chi-square test and t-test

Duration between the date of index and CKD was 4.70 ± 3.41 years for overall, 4.74 ± 3.36 years for Constipation, and 4.67 ± 3.46 years for non- Constipation (t-test *p* = 0.31)

**Fig. 2:**
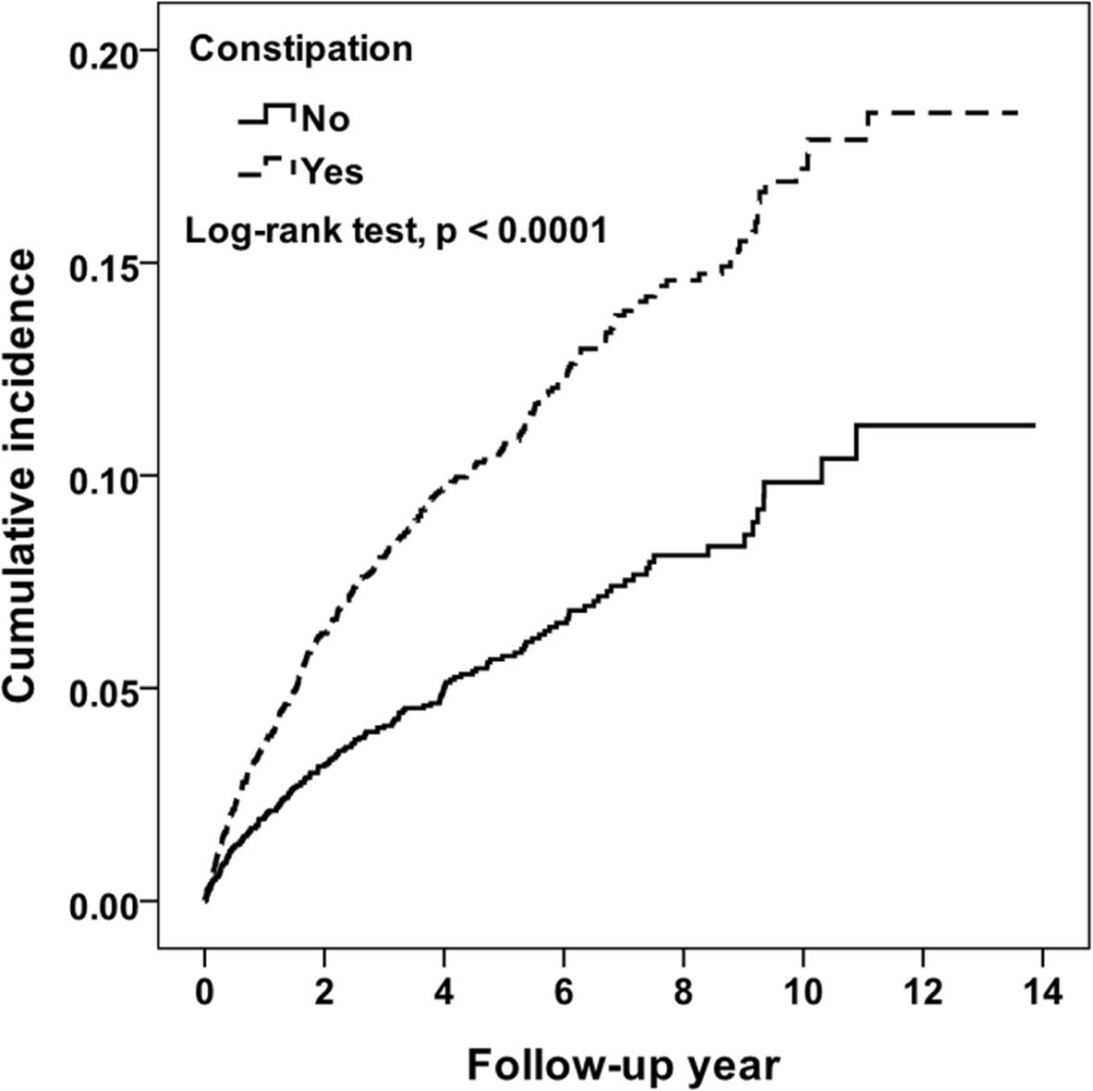
Cumulative incidence for ESRD between patients with and without constipation

In follow-up to the end of 2013, 371 patients (9.5%) experienced ESRD in those with constipation as compared to 182 (4.7%) in those without constipation, respectively (Table 2). Over the 13-year period, the incidences of ESRD per 1000 person-years were 22.9 for constipation group and 12.2 for non-constipation group. Figure 2 showed that there was statistically significant difference for cumulative incidences for ESRD between the patients with and without constipation (*P* < 0.0001). Cox proportional hazard models with a time-dependent variable revealed an adjusted HR of 1.90 (95% CI, 1.60–2.27) in the CKD patients with constipation (Table 2). Compared to the CKD patients without constipation, adjusted HR for the CKD patients with laxatives < 33, 33–197 and ≥ 198 days per year were 0.45 (0.31–0.63), 1.85 (1.47–2.31) and 4.41 (3.61–5.39) respectively. Adjusted HR for the CKD patients with laxatives ≤ 1 type and > 1 type during the study period were 2.08 (1.76–2.50) and 1.53 (1.19–1.95), respectively, compared to the non-constipation group.

**Table 2:** Incidence and hazard ratio for ESRD and associated risk factor

Variable	N	ESRD events	Person-years	*Rate	HR (95% CI)	p-value
Constipation						
No	3909	182	14906	12.21	Ref.	
Yes	3909	371	16232	22.86	1.90 (1.60–2.27)	< 0.0001
Constipation, duration						
No	3909	182	14906	12.21	Ref.	
With Laxatives, < 33 days per year	1291	36	7008	5.14	0.45 (0.31–0.63)	< 0.0001
With Laxatives, 33–197 days per year	1291	123	5504	22.35	1.85 (1.47–2.31)	< 0.0001
With Laxatives, ≥ 198 days per year	1327	212	3720	56.99	4.41 (3.61–5.39)	< 0.0001
Constipation, numbers of types of Laxatives						
No	3909	182	14906	12.21	Ref.	
With Laxatives, ≤ one type during the study period	2844	277	10882	25.45	2.08 (1.76–2.50)	< 0.0001
With Laxatives, > one type during the study period	1065	94	5349	17.57	1.53 (1.19–1.95)	0.0008

*Rate: ESRD events per 1000 person-years

## Discussions

To investigate the impact of kidney-gut axis, our study compared the difference of occurrence of ESRD between CKD patients with newly developed constipation and without constipation. The baseline of demographic and socioeconomic status was matched by propensity scores. The well-known comorbidities related to the progression of CKD and the nephrotoxic medications were also included and matched. Furthermore, the concomitant diseases and medications that might cause constipation were also added and matched. The 2 groups, CKD with constipation and CKD without constipation, were fairly matched and then followed longitudinally from 2000 to 2013. The ESRD events was 371, or 22.9 per 1000 person-year, in CKD with constipation and 182, or 4.7 per 1000 person-year, in CKD without constipation respectively. The cumulative incidence for ESRD in CKD with constipation was significantly higher than those without. The adjusted HR for CKD with constipation was 1.90 (95% CI, 1.60–2.27). The result showed de novo constipation in CKD patients increased the risk of the development of ESRD. To clarify the relationship between CKD and constipation, we further analyzed the incidence of ESRD in constipation patients with different duration of laxatives use per year and different numbers of types of laxatives. Compared to the CKD patients without constipation, adjusted HR for the CKD patients with laxatives < 33, 33–197 and ≥ 198 days per year were 0.45 (0.31–0.63), 1.85 (1.47–2.31) and 4.41 (3.61–5.39) respectively. For mild constipation, especially those who nearly did not need laxatives (laxatives < 33 days), the HR (0.45) of ESRD did not increase. However, for obvious constipation, with laxative use more than 33 days per year, the HR (1.85 for 33–197 and 4.41 for ≥198) of ESRD significant higher than non-constipation group. For those with even longer duration of laxatives use, more than 198 days per year, the HR (4.41) was higher than those with laxative use between 33 and 197 days per year (1.85). The result showed the mild constipation, with nearly no laxatives needed, would not increase the risk of ESRD; however, the obvious constipation, with laxatives use more than one month per year, would increase the risk of ESRD. Furthermore, more severe constipation, with laxatives use more than 6 months per year, would bring even higher risk of ESRD. There seemed to be a dose-effect relationship between CKD and constipation, which consolidated our hypothesis. The adjusted HR for the CKD patients with laxatives less than or equal to one type (≤ 1 type) and more than one type (> 1 type) during the study period were 2.08 (1.76–2.50) and 1.53 (1.19–1.95), respectively, compared to the non-constipation group. Both ≤ 1 type and > 1 type laxatives showed significantly increased HR, but the > 1 type did not have higher HR than ≤ 1 type. Combining the analysis above, the duration of laxative use should account more in the risk of ESRD than the different numbers of types of laxatives use.

The main limitation of this study is that we cannot rule out the possibility of residual confounding by unmeasured factors such as lifestyle and behavioral factors such as diet, smoking, alcohol drinking and exercise. Otherwise, we tried to reduce the confounders related to the progression of CKD and the development of constipation. To reduce the confounders related to the progression of CKD, several associated comorbidities, including acute coronary syndrome, diabetes, hypertension, hyperlipidemia, COPD and cerebrovascular disease, were added to the propensity score-matching models (Table 1). Previous use of NSAIDs and analgesic drugs other than NSAIDs were also added to matching. To reduce the confounders related to the development of constipation, the concomitant diseases and related medications which may cause constipation were also added into the matching. We tried to find if the de novo constipation per se increased the risk of ESRD in CKD patients.

Constipation status and severity was reported as a risk factor of incident CKD and ESRD [24]. It suggested healthy people needed to monitor their defecation status to maintain kidney health. Our study focused on the CKD people and suggested they should maintain good bowel habit to avoid de novo constipation which might precipitate the CKD progression. Moreover, even the constipation could be controlled by the laxatives, but the erroneous gut microbiome, that caused the negative effect to kidney via kidney-gut axis, might not be changed. Therefore, some other interventions, like the use of probiotics and prebioitics [25–27], smart bacteriae [28], and high-fiber diet [29], or the application of toxin-absorbing agents [30, 31] should be considered to correct this error. It is also relevant to define the specific microbiome profiles associated with CKD and ESRD to give the precise therapy. Future studies should explore the profiles of microbiome in CKD and the interaction between them.

## Conclusions

Our longitudinal nationwide study showed that de novo constipation in CKD patients was a risk factor in progression to ESRD. The obvious constipation, with laxatives needed, increased the risk. The more severe constipation, with the longer duration of laxatives needed, increased the risk further. The patency issue of bowel should not be ignored in CKD patients. Dietary modification and some other interventions should be tried to avoid constipation. Avoidance or even shortened intervals of constipation might be helpful to lessen the progression of CKD.

## Additional file

Additional file 1: **Table S1.** Diseases and corresponding ICD-9-CM codes. **Table S2.** Anatomical Therapeutic Chemical codes of drugs used concomitantly by patients during the study period. (DOCX 21 kb)

## Data Availability

The LHID 2000 is a medical claims database by the NHI of Taiwan. The use of NHIRD is limited to research purposes only. Only citizens of the Republic of China who fulfill the requirements of conducting research projects are eligible to apply for the National Health Insurance Research Database (NHIRD). Applicants must follow the Computer-Processed Personal Data Protection Law (http://www.winklerpartners.com/?p=987) and related regulations of National Health Insurance Administration and NHRI (National Health Research Institutes), and an agreement must be signed by the applicant and his/her supervisor upon application submission. The datasets generated and analysed during the current study are available from the authors on reasonable request.
