# Supplementary material for: Association of Constipation with risk of end-stage renal disease in patients with chronic kidney disease
Source: BMC Nephrol. 2019 Aug 5;20:304. doi: 10.1186/s12882-019-1481-0 (PMC6683335; doi:10.1186/s12882-019-1481-0)
Supplement: Supplementary file 1 — Table S1. Diseases and corresponding ICD-9-CM codes. Table S2. Anatomical Therapeutic Chemical codes of drugs used concomitantly by patients during the study period. (DOCX 21 kb) [file 12882_2019_1481_MOESM1_ESM.docx]

Supplementary S1. Diseases and corresponding ICD-9-CM codes

| Disease | Corresponding ICD-9-CM codes |
| --- | --- |
| Chronic kidney disease | 016.00, 095.40, 189.00, 189.90, 223.00, 236.91, 250.40, 271.40, 274.10, 283.11, 403.01, 403.11, 403.91, 404.02, 404.12, 404.92, 404.03, 404.13, 404.93, 440.10, 442.10, 447.30, 572.40, 580.00, 580.40, 580.80, 580.81, 580.89, 580.90, 581.00, 581.10, 581.20, 581.30, 581.80, 581.81, 581.89, 581.90, 582.00, 582.10, 582.20, 582.40, 582.80, 582.80, 582.89, 582.90, 583.00, 583.10, 583.20, 583.40, 583.60, 583.70, 583.80, 583.81, 583.89, 583.90, 584.00, 584.50, 584.60, 584.70, 584.80, 584.90, 585.00, 586.00, 587.00, 588.00, 588.10, 588.90, 591.00, 642.10, 642.20, 753.12, 753.13, 753.14, 753.15, 753.16, 753.17, 753.19, 753.20, 794.40 |
| Co-morbidity |  |
| Acute coronary syndrome | 410, 411, 412 |
| Diabetes | 250 |
| Hypertension | 401-405 |
| Hyperlipidemia | 272 |
| Chronic obstructive pulmonary disease | 491, 492, 496 |
| Cerebrovascular disease (Inpatient) | 430-438 |
| Gastrointestinal tract cancer (Registry for Catastrophic Illness Patient Database) | 150-159 |
| Inflammatory bowel disease | 555-556 |
| Hypothyroidism | 244 |
| Parkinson's disease | 332 |
| Mytonic dystophy | 359.21 |
| Chronic renal failure under regular dialysis (Registry for Catastrophic Illness Patient Database) |  |
| End-stage renal disease | 585 |
| Hypertensive heart or renal disease with renal failure | 403.01, 403.11, 403.91, 404.02, 404.03, 404.12, 404.13, 404.92, 404.93 |

Abbreviation: ICD-9-CM, International Classification of Disease, 9^th^ Revision, Clinical Modification

Supplementary S2. Anatomical Therapeutic Chemical codes of drugs used concomitantly by patients during the study period

| **Drug type** | **ATC classification system codes** |
| --- | --- |
| **NSAIDs** | M01AA, M01AB, M01AC, M01AE, M01AG, M01AX |
| **Analgesic drugs other than NSAIDs** | |
| COXⅡ inhibitor | M01AH |
| Acetaminophen | N02BE01 |
| Aspirin | N02BA01 |
| **Antihypertensive drugs** |  |
| Diuretics | C03 |
| Beta-blockers | C07 |
| Calcium channel blockers | C08、C07FB、C09BB、C09DB |
| **Aluminum antacids** | A02AB |
| **Antiarrythmics** | C01B |
| **Anticonvulsants** | N03 |
| **Antidepressants** | N06A |
| **Antidiarrheals** | A07F、A07X |
| **Antihistamines** | R06A |
| **Antispasmodic** | A03C、A03D、A03E、A02AG、A03AC、N02AG |
| **Calcium supplement** | A12A |
| **Iron supplement** | B03A、A11AA01 |
| **Opioids** | N02A |
| **Serotonin (5HT3) antagonists** | A04AA |
